# Changes of Soil Bacterial Diversity as a Consequence of Agricultural Land Use in a Semi-Arid Ecosystem

**DOI:** 10.1371/journal.pone.0059497

**Published:** 2013-03-20

**Authors:** Guo-Chun Ding, Yvette M. Piceno, Holger Heuer, Nicole Weinert, Anja B. Dohrmann, Angel Carrillo, Gary L. Andersen, Thelma Castellanos, Christoph C. Tebbe, Kornelia Smalla

**Affiliations:** 1 Institute for Epidemiology and Pathogen Diagnostics - Federal Research Centre for Cultivated Plants (JKI), Braunschweig, Germany; 2 Department of Ecology, Lawrence Berkeley National Laboratory, Berkeley, California, United States of America; 3 Institute for Biodiversity, Johann Heinrich von Thünen-Institut (TI), Braunschweig, Germany; 4 Centro de Investigaciones biologicas del Noroeste, S.C. La Paz, Mexico; U. S. Salinity Lab, United States of America

## Abstract

Natural scrublands in semi-arid deserts are increasingly being converted into fields. This results in losses of characteristic flora and fauna, and may also affect microbial diversity. In the present study, the long-term effect (50 years) of such a transition on soil bacterial communities was explored at two sites typical of semi-arid deserts. Comparisons were made between soil samples from alfalfa fields and the adjacent scrublands by two complementary methods based on 16S rRNA gene fragments amplified from total community DNA. Denaturing gradient gel electrophoresis (DGGE) analyses revealed significant effects of the transition on community composition of *Bacteria*, *Actinobacteria*, *Alpha*- and *Betaproteobacteria* at both sites. PhyloChip hybridization analysis uncovered that the transition negatively affected taxa such as *Acidobacteria*, *Chloroflexi*, *Acidimicrobiales*, *Rubrobacterales*, *Deltaproteobacteria* and *Clostridia*, while *Alpha-, Beta- and Gammaproteobacteria, Bacteroidetes* and *Actinobacteria* increased in abundance. Redundancy analysis suggested that the community composition of phyla responding to agricultural use (except for *Spirochaetes*) correlated with soil parameters that were significantly different between the agricultural and scrubland soil. The arable soils were lower in organic matter and phosphate concentration, and higher in salinity. The variation in the bacterial community composition was higher in soils from scrubland than from agriculture, as revealed by DGGE and PhyloChip analyses, suggesting reduced beta diversity due to agricultural practices. The long-term use for agriculture resulted in profound changes in the bacterial community and physicochemical characteristics of former scrublands, which may irreversibly affect the natural soil ecosystem.

## Introduction

Converting natural land into arable soils results in losses to the landscapes characterized by a typical indigenous flora and fauna. Frequently, terrestrial ecosystem diversity is being reduced by replacing indigenous flora with a few crops. The ecological consequences of such transitions have been addressed in several studies focusing on land degradation [1], [2], [3], losses of macro-biodiversity [4], [5], nutrient exhaustion in soils [3], sustainability [6], [7] and restoration [8]. Soil microorganisms, including protozoa, fungi, bacteria and archaea, are essential for the proper functioning and sustainability of ecosystems [9], [10], [11]. Moreover, a high microbial diversity is assumed to be critical for the stability of ecosystems by providing functional diversity and redundancy [12], [13]. Changes in vegetation as well as intensive agricultural practices were shown to affect soil microbial community composition and activity [14], [15] and soil physicochemical properties [3]. The influence of land use and management on soil microorganisms was addressed in several recent studies [16], [17]. However, the information acquired is still not sufficient as a systematic identification of taxa responding to the transition in land use was not done.The studies investigated soils from various geographic sites in Australia, The Netherlands, and Brazil [16] although a comparison of the results might also be difficult due to the differences in the experimental designs and the resolution level of the methods used.

The Santo Domingo Valley is an agricultural area within the Southern Sonoran Desert, Baja California Sur, Mexico, which is entirely dependent on irrigation water collected from wells. Most of the farmland has been developed during the 1950’s and 60′s with cotton and wheat as the main crops [18]. Since then, other crops have been cultivated, e.g., oat, sorghum, chickpea, maize, and alfalfa. Due to declining water availability and increasing problems with soil salinization, yields have decreased since 1991 [18]. Most of the arable land is bordered by natural sarcocaulescent scrubland, including different crassicaulent plants, succulent cacti, woody trees and shrubs [19]. Some of these areas are utilized for grazing goats [20], [21] and thus might be impacted by fecal depositions. The soils in the Santo Domingo Valley belong to the hyposodic calcisols, which are typical of many semi-arid ecosystems. The two sites selected provided almost ideal conditions to study the effects of agricultural land use on the microbial communities in these ecosystems as agricultural fields were in direct vicinity to the scrubland and the soils were typical of semi-arid deserts.

In the present study, bacterial soil communities from arable fields with alfalfa and the adjacent scrubland at two sites 50 km apart from each other were compared by denaturing gradient gel electrophoresis (DGGE) and PhyloChip analysis of16S rRNA gene fragments amplified from total community DNA to evaluate the influence of land use. Both methods are complementary but have clear differences. DGGE provides information on the relative abundance of all amplified dominant populations and thus is more suitable for comparative analysis of the community composition. In the present study the bacterial community analyses by DGGE was performed at different taxonomic levels, *Bacteria*, *Actinobacteria, Alpha-* and *Betaproteobacteria* in order to analyze not only dominant bacteria. The so-called PhyloChip developed by Brodie et al. [36] offers the potential to detect 8741 operational taxonomic units (OTUs) and the dataset is ideal for identifying taxa containing a high proportion of OTUs with treatment dependent significantly increased or decreased abundance. Therefore, the PhyloChip dataset was used to analyze whether, and if so which bacterial taxa responded to agricultural use, and multivariate statistics was applied to explore the relationship between discriminative soil parameters and responsive taxonomic groups.

## Materials and Methods

### Site and Sampling

Two typical sites in the Santo Domingo valley, Baja California, Mexico (site 1: N25°06′31.5″ W111°32′34.3″, altitude 70; site 2: N25°16′5.2″ W111° 36′ 02.5″, altitude 90; the distance between site 1 and site 2 is ca 50 km; No specific permits were required for the described field studies. The location is not protected in any way. The field studies did not involve endangered or protected species.) were selected. Soil samples were taken on 30 April 2007 from two covers, i.e., a field planted with alfalfa (*Medicago sativa*) and the adjacent natural scrubland. Four replicates per site and cover were taken respectively from plots (1 m×1 m squares) that were at least 20 m apart from each other. After removing the top 1–2 cm soil layer, the soil from 2–15 cm depth was mixed with a shovel and approx. 2 kg of soil was sampled, put into plastic bags and transferred to the laboratory. Within 24 h after sampling, the soils were sieved through a 2 mm mesh and aliquots were used for microbiological and chemical analysis.

### Soil Properties

All soils were analyzed by the certified laboratory at CIBNOR (La Paz, Mexico). Briefly, soil particle size determinations were conducted with the sedimentation method [22], pH was determined in 1∶1 (wt/vol) diluted water suspensions [23], electrical conductivity was determined with a CO150 conductivity meter according to the manufacturer’s instructions (Hach Company, Loveland, CO, USA). Total organic matter was measured using the reduction of potassium dichromate method of Walkley and Black, as described by Nelson and Sommers [24]. Furthermore, ammonium (NH_4_^+^), nitrite and nitrate [25], calcium (Ca^2+^) [26], magnesium (Mg^2+^), phosphate, sulfate [27] were quantified. All soil parameters measured are given in Table 1.

**Table 1:** Physico-chemical characteristics (average ± standard deviation) for soils from different sites and land use.

	Site 1		Site 2	
	alfalfa	scrubland	Alfalfa	scrubland
pH value	8.65±0.12	8.7±0.27	8.62±0.06	8.1±0.64
Electric conductivity [mS cm^−1^]	0.86±0.14	0.59±0.18	1.45±0.33	1.3±1.71
Ca^2+^ [mg kg^−1^]	8.67±2.16	9.89±3.36	10.83±4.56	13.2±14.17
Mg^2+^ [mg kg^−1^]	5.42±1.38	5±0.98	13.34±5.32	6.63±6.46
K^+^ [mg kg^−1^]	4.59±1.62	4.8±1.99	5.09±2.7	4.01±2.14
Na^+^ [mg kg^−1^]* +	33.22±6.34	13.86±9.37	44.56±8.2	26.4±32.55
Phosphate [mg kg^−1^]*** −	5.2±1.33	41.62±19.15	7.21±3.32	30.32±17.97
NH_4_^+^-N [mg kg^−1^]	10.14±4.1	7.44±1.53	10.48±2.17	10.14±3.3
Total nitrogen [mg kg^−1^]	482.39±179.03	450.48±76.27	358.29±130.19	308.65±170.74
Cl^−^ [mg kg^−1^]	37.6±5.28	11.57±7.57	60.23±15.41	65.64±110.05
NO_2_^–^N [mg kg^−1^]	0.05±0.03	0.03±0.03	0.09±0.16	0.03±0.04
NO_3_^–^N [mg kg^−1^]* +	2.62±0.72	0.66±0.5	1.18±1.92	0.23±0.22
Sulphate [mg kg^−1^]*** +	8.35±1.63	4.79±1.33	29.28±6.24	2.97±0.92
Organic matter [% volumetric]* −	0.45±0.06	0.79±0.18	0.44±0.11	0.58±0.32

Two-way ANOVA was used to test for significant differences in soil parameters between land use. The significant level between land use was indicated as

* p<0.05;

*** p<0.001; +: significantly increased in agricultural soils; −: significantly decreased in agricultural soils.

### Total Community (TC) DNA

TC DNA was extracted from 0.5 g of soil after a harsh lysis step (FastPrep FP120 bead beating system, MP Biomedicals, Santa Ana, Carlsbad, CA, USA) by means of the BIO-101 DNA spin kit for soil (Q-Biogene). The DNA was purified using the Geneclean Spin Kit (Q-Biogene) according to the manufacturer’s instructions. Purified TC DNA was stored at −20°C.

### PCR Amplification of 16S rRNA Gene Fragments and DGGE Analysis

Primer sets and PCR conditions employed in this study to amplify *Bacteria* [28], *Actinobacteria* [28], and *Alpha*- and *Betaproteobacteria* [29], [30] and relevant information is provided in Table S1. DGGE of the 16S rRNA gene amplicons was performed according to Gomes *et al.* [31]. The gel was silver-stained according to Heuer *et al*. [32]. DGGE profiles were analyzed by GelCompar 4.5. Dendrograms were constructed by means of unweighted pair group method using arithmetic averages (UPGMA) based on pairwise Pearson correlation indices, which were also subjected to permutation tests with a modified version of PERMTEST software [33]. Box-Whisker plots were generated using R (http://www.R-project.org) based on dissimilarities (1- Pearson correlation indices) between samples within the same treatment.

### PCR Amplification of 16S rRNA Genes and PhyloChip Analysis

TC DNA extracts from three replicates per site and cover were amplified using universal 16S rRNA gene primers (27f 5′- AGAGTTTGATCCTGGCTCAG-3′; 1492r 5′- GGTTACCTTGTTACGACTT-3′) and an 8-temperature gradient PCR. At each temperature, approximately 5 ng of TC DNA was used in 25 µl reactions (final concentrations were 1× Ex Taq Buffer with 2 mM MgCl_2_, 300 nM each primer (27 f and 1492 r), 200 µM each dNTP (TaKaRa), 25 µg bovine serum albumin (Roche Applied Science, Indianapolis, IN, USA), and 0.625 U Ex Taq (TaKaRa Bio, Inc., through Fisher Scientific, Pittsburg, PA, USA)). The amplifications were performed with an iCycler (Bio-Rad, Hercules, CA, USA) as previously described by Weinert *et al.* [34]. PCR products from each annealing temperature (48–58°C) for each sample were combined, concentrated, quantified, and an amount of 500 ng product was applied to each G2 PhyloChip (Second Genome Inc., San Bruno, CA, USA) following previously described procedures [35]. The PhyloChip used in the present study contained approximately 500,000 probes (25-mer oligos) targeting 8,364 bacterial operational taxonomic units (OTUs). An OTU was considered present if more than 90 percent of the probe pairs representing this OTU showed a positive hybridization signals [36].

### Statistical Analysis

An OTU-level report was produced mainly according to Brodie *et al.* [36] (background subtraction, detection and quantification criteria) except for the addition of normalizing array data by the average total array signal intensity [37]. The signal intensities of OTUs called absent were shifted to 1 to avoid errors in subsequent log transformation. Statistical analyses were done with the software package R 2.14 (http://www.r-project.org/).

Discriminative OTUs between the two different land uses were identified by multiple two-way ANOVA of log10-transformed signal intensities for each OTU (unadjusted p<0.05). Groups with a high proportion of OTUs significantly responding to land use were summarized at different taxonomic levels. The influence of land use on the community compositions of different taxonomic groups (from domain to family) was analyzed by a modified test based on five principal components [38]. Principal components analysis (PCA) was performed according to Weinert *et al*. [34] using adjusted (log10- transformed, centered, and standardized) signal intensities of all OTUs belonging to each taxonomic group.

Soil parameters responding to land use were also identified by multiple two-way ANOVA (unadjusted p<0.05). Heatmap analysis was performed based on the adjusted (centered and standardized) values for different soil parameters. To analyze the influence of these discriminative soil parameters on community composition of total bacteria or responsive phyla, redundancy analysis (RDA) was performed using the R add-on package ‘vegan’. A forward selection of soil parameters was applied to avoid using collinear soil parameters in the same constrained ordination model. Only those parameters contributing significantly (p<0.05 via 1000 times permutation tests) to community variation were added to the model.

## Results

### Significant Effects of Land Use on the Bacterial Community Composition Revealed by DGGE Fingerprints

To compare the bacterial community in soils under different land use, 16S rRNA gene fragments of bacteria, *Alphaproteobacteria*, *Betaproteobacteria* and *Actinobacteria* PCR-products amplified from total community DNA of alfalfa or adjacent scrubland soils sampled at two sites were analyzed by DGGE (Figures S1, S2, S3, S4).

**Figure 1:**
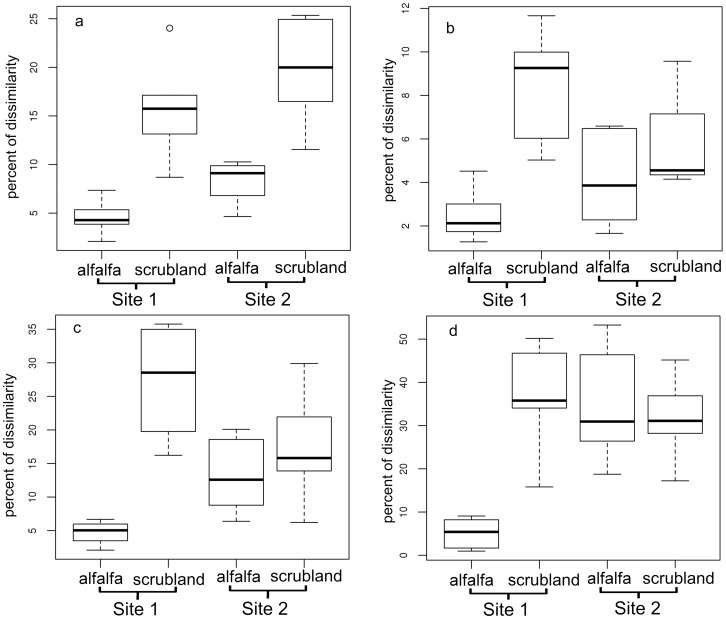
Boxplots of the variation of community structure under same sites and land use based on DGGE profiles for a: *Bacteria*; b: *Actinobacteria*; c: *Alphaproteobacteria*; d: *Betaproteobacteria*.

Pairwise Pearson correlation indices were subjected to permutation tests to determine the significance of the land use effects on the bacterial community structure. A significant effect of land use was found for bacteria and all bacterial groups analyzed for both sites (Table 2). The extent of the influence was dependent on the phylogenetic group analyzed (Table 2). The strongest influence of land use was found for the *Betaproteobacteria*, especially at site 1 (Table 2). In the community profile for *Betaproteobacteria*, a strong band was observed only in all replicates in soils with alfalfa from site 1 (Figure S4). For *Alphaproteobacteria*, the dissimilarities of bacterial community fingerprints of soil under different land use were comparable between both sites. The lowest yet still significant effect of land use was observed for *Actinobacteria* (Table 2). Significant differences in community composition between the two study sites were found mainly for alfalfa growing soils as opposed to scrubland soils (Table 2). Compared to the effects of land use, the influence of different sites on community composition was smaller except for *Betaproteobacteria* (Table 2), probably still due to the strong bands for alfalfa soils from site 1 (Figure S4). For bacteria including all bacterial subgroups analyzed, Box-Whisker plots revealed that the variability of the bacterial community compositions among replicates was generally lower for alfalfa soils than the scrubland soil, except for *Betaproteobacteria* at site 2 (Figure 1). The lowest variability was found for alfalfa soils from site 1 (Figure 1). In conclusion, a significant and taxonomic group-dependent effect of land use was observed for all four targeted phylogenetic groups. Variation in community composition for soils under arable farmland use generally was lower than that from scrubland sites.

**Table 2:** Percent dissimilarity of microbial DGGE fingerprints of different taxa for soils compared between alfalfa and scrubland or between site 1 and site 2.

		*Bacteria*	*Alpha-proteobacteria*	*Beta-proteobacteria*	*Actinobacteria*
Land use	Site 1	25*	20.3*	68.6*	2.7*
	Site 2	13.8*	25*	31.3*	3.7*
					
Site	Alfalfa	10.3*	11.8*	61.5*	3.7*
	Scrubland	3.8	4.9	5.8	2.8*

* Significant (p<0.05) difference in community fingerprints between treatments as revealed by permutation tests.

### Taxa Responsive to Land Use Determined by PhyloChip Analysis

PhyloChip hybridizations were used to detect bacterial taxa with significant responses to land use. A total of 2,243 OTUs belonging to 44 phyla was detected (Table S2). The bacterial richness in terms of the number of detected OTUs was significantly (p = 0.05) higher for the arable field soils than for the scrubland soils.

OTUs responding to land use were identified by multiple two-way ANOVA and only taxa with a high proportion (>18% which is much higher than the unadjusted p-value) of discriminative OTUs were summarized in Table 3. Compared to the soil from the scrublands, 13% of the OTUs (295 OTUs) were more abundant in soil from alfalfa fields, while more OTUs (402 accounting for 18%) were less abundant (Table 3), suggesting few taxa probably enriched in the alfalfa fields. A large proportion (more than 25%) of the OTUs belonging to the phyla of *Acidobacteria*, *Chloroflexi*, *Spirochaetes*, *Verrucomicrobia* and *Gemmatimonadetes* were significantly less abundant in alfalfa soils (Table 3), suggesting that the transition of scrubland soil into arable soils caused severe effects on the abundance of OTUs affiliated to these phyla. A negative effect also was observed for the class of *Clostridia* (*Firmicutes*), of which 29% of the OTUs detected had significantly lower signal intensities in the alfalfa soils (Table 3). The influence of land use on the phyla *Proteobacteria* and *Actinobacteria* was more complex as more than 40% of the OTUs belonging to three order*s* of the *Deltaproteobacteria* (*Desulfobacterales*, *Desulfovibrionales* and *Syntrophobacterales*) were less abundant while many taxa belonging to *Alphaproteobacteria* (*Rhizobiales*: *Rhizobiaceae, Phyllobacteriaceae*; *Sphingomonadales; Rhodobacterales), Betaproteobacteria* (*Burkholderiales; Comamonadaceae*) and *Gammaproteobacteria (Alteromonadales; Pseudomonadales: Pseudomonadaceae*) contained a high proportion of OTUs (>18%) that were more abundant in alfalfa soils (Table 3). An exception for *Gammaproteobacteria* was the order of *Legionellales*, of which ca. 56% of the OTUs were more abundant in the scrubland soils (Table 3). Interestingly, for *Rhodobacterales* (*Alphaproteobacteria*) 40% of the OTUs were more abundant in alfalfa soils, while 20% of the OTUs were lower compared to scrubland soils (Table 3). A high proportion (>33%) of the OTUs belonging to two orders of *Actinobacteria* (*Acidimicrobiales* and *Rubrobacterales*) had lower relative abundance in natural scrublands compared to alfalfa soils. Several families in the order of *Actinomycetales* (*Microbacteriaceae, Micromonosporaceae, Micrococcaceae,* and *Cellulomonadaceae*) had more OTUs with significantly higher abundance in alfalfa soils than in the scrubland soils (Table 3). In contrast to these families, *Mycobacteriaceae* and *Pseudonocardiaceae* decreased in relative abundance with change in land use (Table 3).

**Table 3:** Taxa and numbers (percent of all detected OTU belonging to each taxon) of OTUs significantly (unadjusted P<0.05) enriched in alfalfa or scrubland soil as identified by two-way ANOVA based on PhyloChip.

Phylum	Class	Order	Family	Alfalfa	Scrubland
*Proteobacteria*	*Alphaproteobacteria*	*Rhizobiales*	*Rhizobiaceae*	14 (77.8%)	0 (0.0%)
			*Phyllobacteriaceae*	9 (60.0%)	0 (0.0%)
		***Sphingomonadales***		9 (18.0%)	1 (2.0%)
		***Rhodobacterales***		20 (40.0%)	10 (20.0%)
	*Betaproteobacteria*	*Burkholderiales*	*Comamonadaceae*	36 (62.1%)	0 (0.0%)
	*Gammaproteobacteria*	*Alteromonadales*		15 (26.3%)	2 (3.5%)
		*Pseudomonadales*	*Pseudomonadaceae*	21 (53.8%)	1 (2.6%)
		*Legionellales*		1 (9.1%)	6 (54.5%)
	*Deltaproteobacteria*	*Desulfobacterales*		0 (0.0%)	19 (63.3%)
		*Desulfovibrionales*		0 (0.0%)	8 (50.0%)
		*Syntrophobacterales*		0 (0.0%)	6 (42.9%)
*Firmicutes*	*Clostridia*			6 (3.4%)	51 (28.8%)
***Actinobacteria***	***Actinobacteria***	*Acidimicrobiales*		1 (5.6%)	6 (33.3%)
		***Actinomycetales***	*Microbacteriaceae*	15 (65.2%)	0 (0.0%)
			***Micromonosporaceae***	6 (27.3%)	2 (9.1%)
			*Mycobacteriaceae*	0 (0.0%)	16 (76.2%)
			*Micrococcaceae*	13 (76.5%)	0 (0.0%)
			***Nocardiaceae***	2 (12.5%)	1 (6.3%)
			*Pseudonocardiaceae*	1 (8.3%)	4 (33.3%)
			*Cellulomonadaceae*	7 (63.6%)	0 (0.0%)
		***Rubrobacterales***		0 (0.0%)	10 (50.0%)
*Acidobacteria*	*Acidobacteria*	*Acidobacteriales*	*Acidobacteriaceae*	0 (0.0%)	21 (56.8%)
*Bacteroidetes*				22 (18.2%)	10 (8.3%)
*Chloroflexi*				1 (2.1%)	25 (53.2%)
*Spirochaetes*				0 (0.0%)	12 (30.0%)
*Verrucomicrobia*				0 (0.0%)	13 (48.1%)
*Gemmatimonadetes*				0 (0.0%)	6 (66.7%)
Bacteria (Total)				295 (13.2%)	402 (17.9%)

All listed taxa have significantly different community structure between land uses except for *Nocardiaceae*. Bold text: Taxa with significantly different community structure between sites.

A modified test based on the first five principal components [38] was applied to study the effect of land use on bacterial community composition of the taxonomic groups listed in Table 3. It revealed and confirmed dramatic differences in the abundance of specific bacterial community members in response to land use. All the taxonomic groups listed in Table 3 had significantly different community compositions, except for *Nocardiaceae* (p = 0.07) (Table 3). As already observed with the DGGE analyses, the variation in the bacterial community composition was higher in the soils from scrubland than from alfalfa fields (Figure S5). Compared to the land use, the two different locations had less influence on the community composition. Effects of the site were only detected for a few taxonomic groups such as the *Sphingomonadales, Rhodobacterales, Rubrobacterales* and *Actinomycetales*. These orders, however, were significantly different in their community composition between the two sites (Table 3).

### Correlation of Land Use Responsive Taxa with Land Use Dependent Soil Parameters

Two-way ANOVA of soil physicochemical parameters revealed that the transition of scrubland into arable land resulted in a significantly increased concentration of sulphate, sodium, salinity, and the ratio of nitrate to total-N. The phosphate concentration and organic matter content were significantly lower in agricultural soils (Table 1).

**Figure 2:**
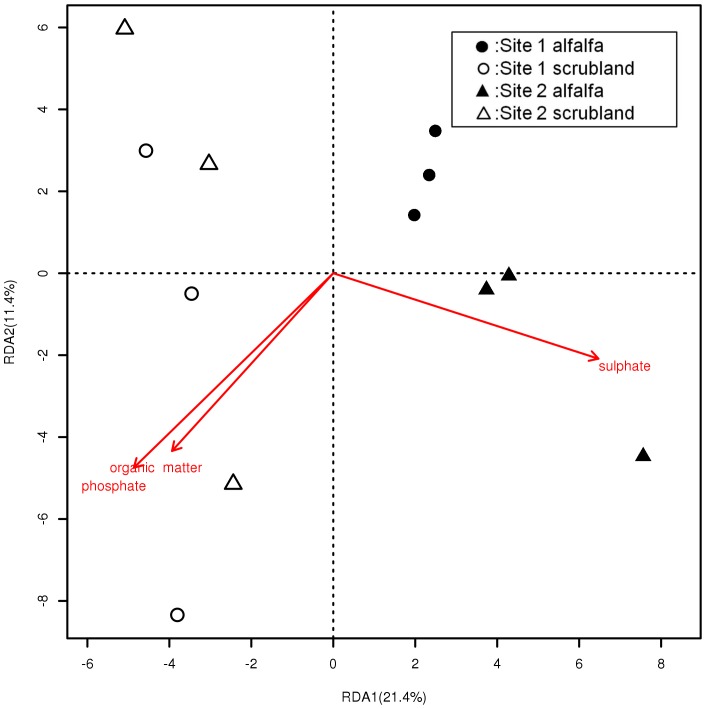
Redundancy analysis of the effect of discriminative soil parameters on bacterial communities using the PhyloChip data. Numbers in brackets indicate the percent of the total variance explained by each axis. Only these soil parameters which significantly (p<0.05 by 1000 times permutation tests) explained the bacterial community variation was shown.

Redundancy analysis was performed to find the correlation between discriminative physicochemical characteristics and variation in the community composition of phyla identified by PhyloChips with significant response to land use. Sulphate and phosphate (phosphate positively collinear with organic matter content) were the main factors that jointly influenced the bacterial community composition (Figure 2). In total 33% of the variation of the bacterial community could be significantly explained by sulphate and phosphate concentrations. Each factor (sulphate, phosphate and organic matter) independently could explain 17% to 21% of the variation. Sulphate and phosphate also explained a considerable amount of the variation within sub-communities of *Proteobacteria* (33%), to which most detected OTUs were affiliated (data not shown) (Figure S6). Discriminative soil parameters also explained a large amount of the variation within *Firmicutes* (19% variation explained by sulphate; Figure S7), *Actinobacteria* (40% explained by phosphate and sulphate; Figure S8), *Acidobacteria* (30% explained by nitrate; Figure S9), *Bacteroidetes* (16% explained by nitrate; Figure S10), *Chloroflexi* (30% explained by nitrate; Figure S11), *Verrucomicrobia* (24% explained by phosphate; Figure S12) and *Gemmatimonadetes* (49% explained by sulphate and sodium; Figure S13). None of these discriminative soil parameters explained the variation within *Spirochaetes,* which appeared to be linked to the pH values of the soils (explaining 32% variation; Figure S14). In general, a considerable amount of variation in the community composition of phyla responding to land use changes could be significantly explained by discriminative soil parameters, suggesting that these soil parameters and the bacterial community structure are strongly connected.

## Discussion

Both DGGE and PhyloChip analysis revealed that the community composition of bacteria at various taxonomic levels differed significantly between the arable soils and the adjacent scrubland soils. The methods used to analyze the effects of the transition in land use were complementary but have clear differences which prevented the use of similar statistical analysis methods to analyze the datasets. In contrast to the DGGE fingerprints that are assumed to reflect the relative abundance of the dominant bacterial populations, the fluorescent signals detected after PhyloChip hybridizations do not necessarily reflect the relative abundance of OTUs. The strength of the PhyloChip approach is that the fluorescence signals of each OTU can be compared horizontally between treatments and thus allows to identify taxa with a high proportion of OTUs with treatment dependent changes in abundance. However, both methods used in this study to analyze PCR-amplified 16S rRNA gene fragments lead to the same conclusions: A strong and significant effect of land use and a higher variability of the community structure was observed for samples from scrubland (likely due to the absence of mixing). Furthermore, compared to the effect of land use, the effect of the site was less pronounced.

Tillage is probably one of the major forces driving shifts in the soil microbial community structure. In addition, irrigation, fertilization, and/or application of agrochemicals have been shown to affect the bacterial community structure [14], [39]–[42]. The minor differences found between the arable soils from site 1 and site 2 may be linked to differences in their previous cropping history and agricultural management, which were most likely not identical. In general, the conversion of scrubland into agricultural land is associated with the replacement of a diverse, indigenous, highly adapted vegetation of the semi-arid desert with a few crop plants. Plant effects on soil microbial communities have been frequently observed in the rhizosphere, which refers to the soil directly influenced by root exudates [30], [43]. The influence of plant species varied between different studies, in some of them it was regarded to be the major factor shaping the microbial community structure [44]–[49], while in others only a minor influence of plant species and vegetation composition on the soil bacterial community composition was observed [50]. However, only a few studies suggested long-term effects of plants on microbial communities in bulk soils [16]. Long-term agricultural use impacts soil physicochemical characteristics [3], [16], [17], and thus probably alters the composition and properties of biogeochemical interfaces in soils [51].

The present study showed that agricultural use impacted several bacterial phyla in the soils of the sites studied, e.g., *Acidobacteria*, *Chloroflexi*, *Spirochaetes*, *Verrucomicrobia*, *Gemmatimonadetes*, *Deltaproteobacteria* (*Proteobacteria*), *Acidimicrobiales* (*Actinobacteria*) and *Rubrobacterales* (*Actinobacteria*). *Acidobacteria* have been found to be dominant in several soils [52] though often they are difficult to cultivate. In the study by Bisette *et al.* [16], the proportion of *Acidobacteria* was found increased in grassland soils compared to soils under agricultural use at one site in Australia. Compared with agricultural soils, their relative abundance was also reported to be higher in forest, desert or prairie soils [52], [53]. The proportion of *Acidobacteria* was reported to be significantly lower in nutrient-rich rhizosphere than in bulk soil, confirming their oligotrophic lifestyle [54]. Under dry conditions, the net primary productivity of plants is controlled by water [55], [56]. Additional water supply in the alfalfa fields enhanced plant growth and probably elevated the extent of plant exudates into soil over a time period of about 50 years. Recently the relative abundance of *Acidobacteria* was found to be negatively correlated with the level of nitrogen input (fertilizer) [57], which in general increases the net productivity of vegetation. *Chloroflexi* also was reported to prevail in nutrient poor soils [52], [58] and other oligotrophic ecosystems such as soils from high-elevation regions where vegetation is patchy [59], alpine tundra soil [60] or hyperarid polar desert soil [61]. A few bacteria belonging to *Chloroflexi* which could be retrieved from soil had very slow rates of growth and mini-colony formation [62], [63] which are typical characteristics of oligotrophic organisms. In accordance with the present study, Fierer *et al*. [57] showed that the relative abundance of *Chloroflexi* was also lower in the plots with high levels of nitrogen input.

In the present study, the physicochemical analysis of the soils done for four independent replicates per site and treatment revealed that the concentration of sulphate (probably due to fertilization) was higher in alfalfa soils. Sulphate can be used as terminal electron acceptor by some anaerobic bacteria. However, a high proportion of OTUs belonging to *Desulfobacterales*, *Desulfovibrionales*, *Syntrophobacterales* or *Clostridia* had significantly lower abundances in the alfalfa soils compared to scrubland soils. By and large, bacteria belonging to these taxonomic groups are strict anaerobes [64] and play an important role in anaerobic carbon cycling in wet terrestrial ecosystems such as rice fields and wet lands [65,66).Furthermore, tilling soils also affects soil aeration [67] and can elevate the activity of aerobic microbes [14]. Notably, in the present study, a significant loss of organic matter also was observed in the agricultural soils and as soil organic matter is an important binding agent of soil particles into aggregates [68], presumably lowering gas diffusion, this may have contributed to lower sulphate-reducing populations in these desert agricultural soils.

High proportions of OTUs belonging to the *Bacteroidetes* or the *Alpha-, Beta-* and *Gammaproteobacteria* as well as several families of *Actinobacteria* were more abundant in alfalfa soils. The higher abundance of *Pseudomonas* (50% of the OTUs with significantly higher signal intensities) in the agricultural soils was also confirmed by the detection of *Pseudomonas*-specific *gacA* genes [46]. Amplicons of the *gacA* gene fragment were obtained only for TC DNA from alfalfa soils, not from scrubland soil (data not shown). *Betaproteobacteria* and *Bacteroides* were considered to contain copiotrophic taxa as their abundance has been positively correlated with carbon mineralization rate and carbon availability [69]. Several studies reported enrichments of *Alphaproteobacteria* [69], *Gammaproteobacteria* [46,70) or *Actinobacteria* [29,43) in the rhizosphere where carbon availability is increased due to root exudates. The number of OTUs detected by PhyloChip analysis was significantly lower in the soil from scrubland than alfalfa field. In general, this finding is in agreement with other studies, in which agriculture or low plant diversity (often a direct result of converting natural land into agricultural use) did not necessarily lead to a reduction of the bacterial diversity detected [48], [71]–[73]. However, the effects of agricultural practices on soil bacterial richness remain to be explored more fully, perhaps at a finer resolution than was obtainable in this study. Firstly, total bacterial species richness in soils is difficult to assess as many populations occur at low abundance [74], [75], [76]. Secondly, a high proportion of OTUs belonging to *Acidobacteria*, *Chloroflexi*, *Spirochaetes*, *Verrucomicrobia*, *Gemmatimonadetes* were more abundant in the scrubland soils, suggesting that these taxa were better adapted to those soils. Compared to *Proteobacteria*, *Actinobacteria* and *Firmicutes,* many fewer OTUs belonging to these taxa were available during probe design. Therefore, bacterial richness of these phyla in the scrubland soil possibly was underestimated.

In summary, although investigated only at two sites that were assumed to be representative for this type of ecosystem, we could show that the use of scrublands for agriculture caused profound changes in the soil bacterial community structure and physicochemical characteristics. Soil parameters that differed between land uses were highly correlated with the community composition of taxa responding to land use. Several, most likely oligotrophic or anaerobic taxa were negatively affected by the change, in contrast, populations with a potentially copiotrophic lifestyle profited and were enhanced in the agricultural soils.

## Supporting Information

Figure S1 **Bacterial DGGE profiles for soils from different sites and land use.** *: samples not included in this study. M: bacterial standard for DGGE electrophoresis.
(TIF)

Figure S2 **Actinobacterial DGGE profiles for soils from different sites and land use.** *: samples not included in this study. M: bacterial standard for DGGE electrophoresis.
(TIF)

Figure S3 **Alphaproteobacterial DGGE profiles for soils from different sites and land use.** *: samples not included in this study. M: bacterial standard for DGGE electrophoresis.
(TIF)

Figure S4 **Betaproteobacterial DGGE profiles for soils from different sites and land use.** *: samples not included in this study. M: bacterial standard for DGGE electrophoresis.
(TIF)

Figure S5 **Principal component analysis of PhyloChip data for soils from different sites and land use.** The first and second principal components explain 31% and 16% of total variance.
(TIF)

Figure S6 **Redundancy analysis of the effect of discriminative soil parameters on the communities of** ***Proteobacteria*** **using the PhyloChip data.** Numbers in brackets indicate the percent of the total variance explained by each axis. Only these soil parameters which significantly (p<0.05 by 1000 times permutation tests) explained the proteobacterial community variation are shown.
(TIFF)

Figure S7 **Redundancy analysis of the effect of discriminative soil parameters on the communities of** ***Firmicutes*** **using the PhyloChip data.** Numbers in brackets indicate the percent of the total variance explained by each axis. Only the soil parameter which significantly (p<0.05 by 1000 times permutation tests) explained the variation of *Firmicutes* community is shown.
(TIFF)

Figure S8 **Redundancy analysis of the effect of discriminative soil parameters on the communities of** ***Actinobacteria*** **using the PhyloChip data.** Numbers in brackets indicate the percent of the total variance explained by each axis. Only these soil parameters which could significantly (p<0.05 by 1000 times permutation tests) explained the actinobacterial community variationare shown.
(TIFF)

Figure S9 **Redundancy analysis of the effect of discriminative soil parameters on the communities of** ***Acidobacteria*** **using the PhyloChip data.** Numbers in brackets indicate the percent of the total variance explained by each axis. Only the soil parameters which significantly (p<0.05 by 1000 times permutation tests) explained the acidobacterial community variation is shown.
(TIFF)

Figure S10 **Redundancy analysis of the effect of discriminative soil parameters on the communities of** ***Bacteroidetes*** **using the PhyloChip data.** Numbers in brackets indicate the percent of the total variance explained by each axis. Only the soil parameters which significantly (p<0.05 by 1000 times permutation tests) explained the community variation of *Bacteroidetes* is shown.
(TIFF)

Figure S11 **Redundancy analysis of the effect of discriminative soil parameters on the communities of** ***Chloroflexi*** **using the PhyloChip data.** Numbers in brackets indicate the percent of the total variance explained by each axis. Only the soil parameter which significantly (p<0.05 by 1000 times permutation tests) explained the community variation of *Chloroflexi* is shown.
(TIFF)

Figure S12 **Redundancy analysis of the effect of discriminative soil parameters on the communities of** ***Verrucomicrobia*** **using the PhyloChip data.** Numbers in brackets indicate the percent of the total variance explained by each axis. Only these soil parameters which significantly (p<0.05 by 1000 times permutation tests) explained the community variation of *Verrucomicrobia* are shown.
(TIFF)

Figure S13 **Redundancy analysis of the effect of discriminative soil parameters on the communities of** ***Gemmatimonadetes*** **using the PhyloChip data.** Numbers in brackets indicate the percent of the total variance explained by each axis. Only these soil parameters which significantly (p<0.05 by 1000 times permutation tests) explained the community variation of *Gemmatimonadetes* are shown.
(TIFF)

Figure S14 **Redundancy analysis of the effect of soil parameters on the communities of** ***Spirochaetes*** **using the PhyloChip data.** Numbers in brackets indicate the percent of the total variance explained by each axis. Only these soil parameters which significantly (p<0.05 by 1000 times permutation tests) explained the community variation of *Spirochaetes* are shown.
(TIFF)

Table S1 **Primers used for DGGE analyses in the present study.**
(DOCX)

Table S2 **Numbers (average ± standard deviation)s of OTU detected for bulk soils from alfalfa field and scrubland at two sites.**
(DOCX)
