# Supplementary figures and images for: Changes of Soil Bacterial Diversity as a Consequence of Agricultural Land Use in a Semi-Arid Ecosystem
Source: PLoS One. 2013 Mar 20;8(3):e59497. doi: 10.1371/journal.pone.0059497 (PMC3603937; doi:10.1371/journal.pone.0059497)

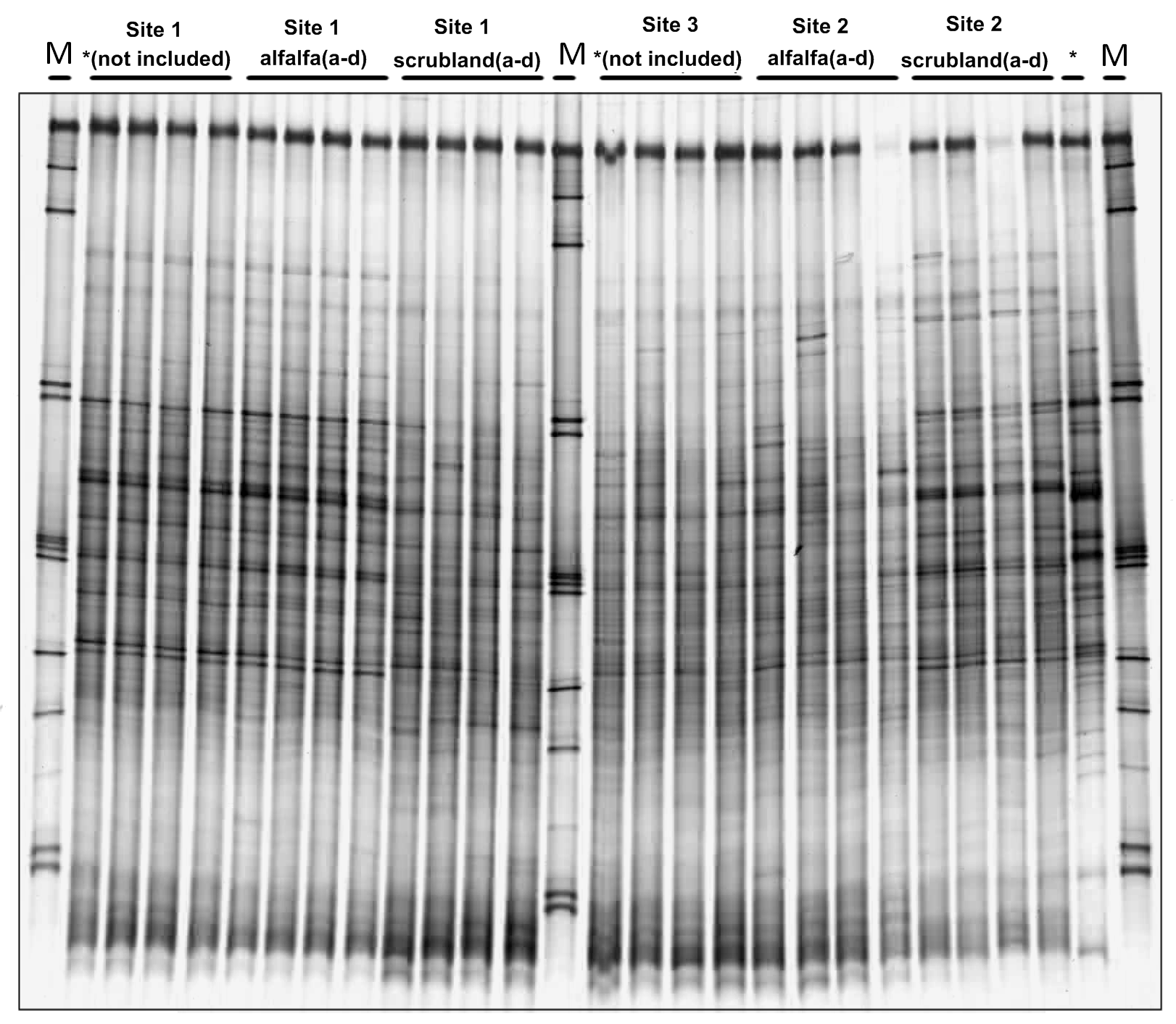

Supplement: Figure S1 — Bacterial DGGE profiles for soils from different sites and land use. *: samples not included in this study. M: bacterial standard for DGGE electrophoresis. (TIF) [file pone.0059497.s001.tif]

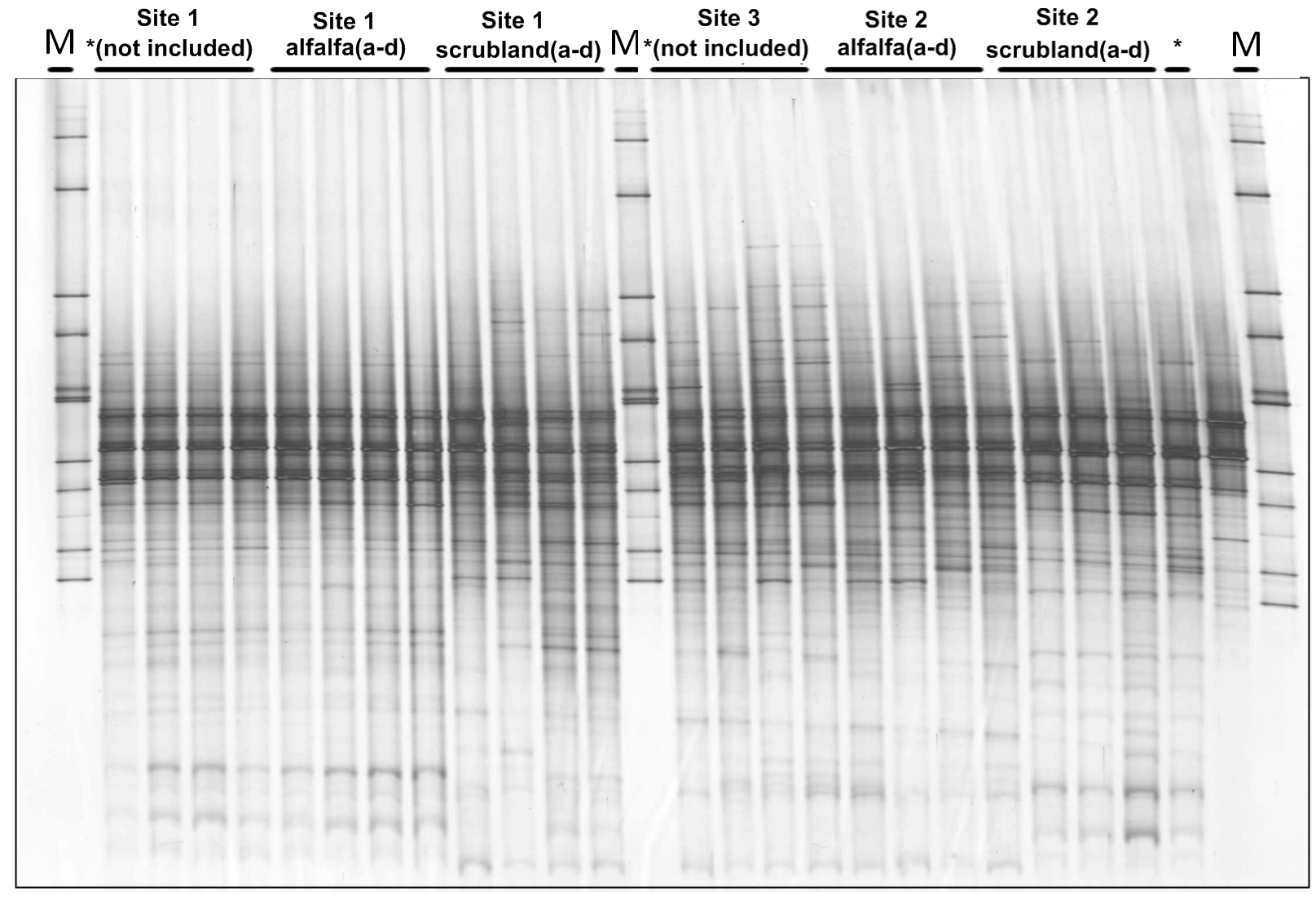

Supplement: Figure S2 — Actinobacterial DGGE profiles for soils from different sites and land use. *: samples not included in this study. M: bacterial standard for DGGE electrophoresis. (TIF) [file pone.0059497.s002.tif]

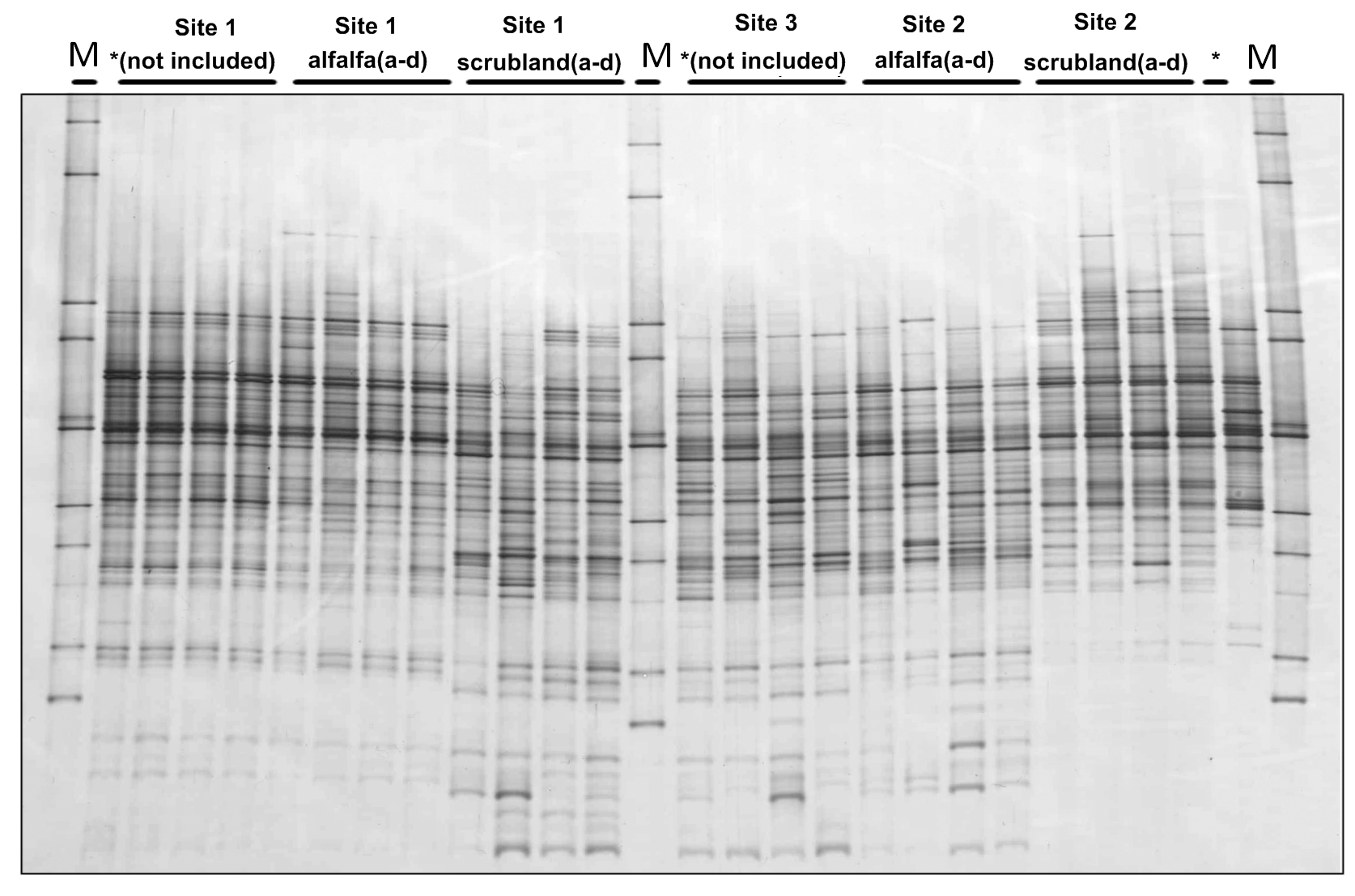

Supplement: Figure S3 — Alphaproteobacterial DGGE profiles for soils from different sites and land use. *: samples not included in this study. M: bacterial standard for DGGE electrophoresis. (TIF) [file pone.0059497.s003.tif]

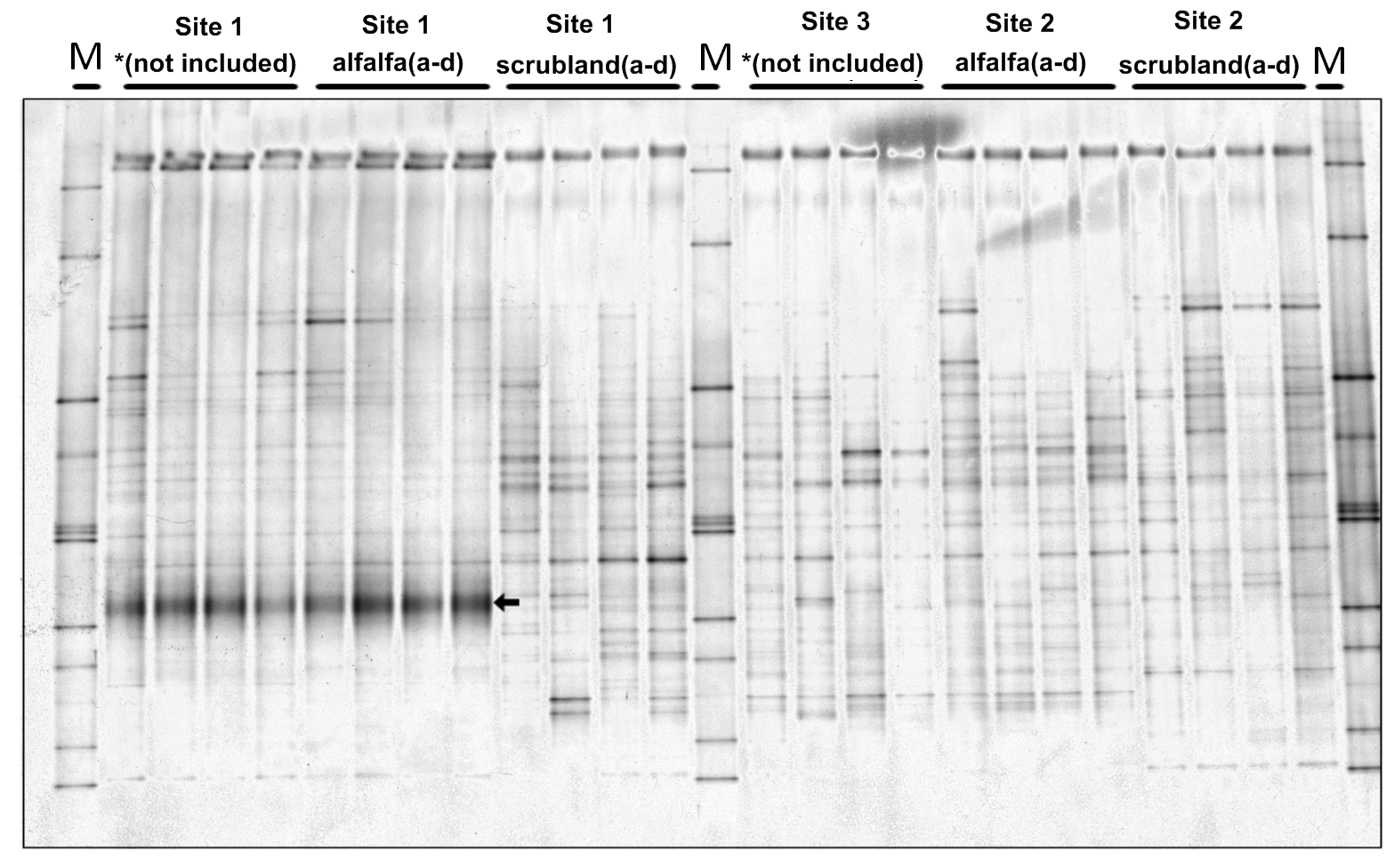

Supplement: Figure S4 — Betaproteobacterial DGGE profiles for soils from different sites and land use. *: samples not included in this study. M: bacterial standard for DGGE electrophoresis. (TIF) [file pone.0059497.s004.tif]

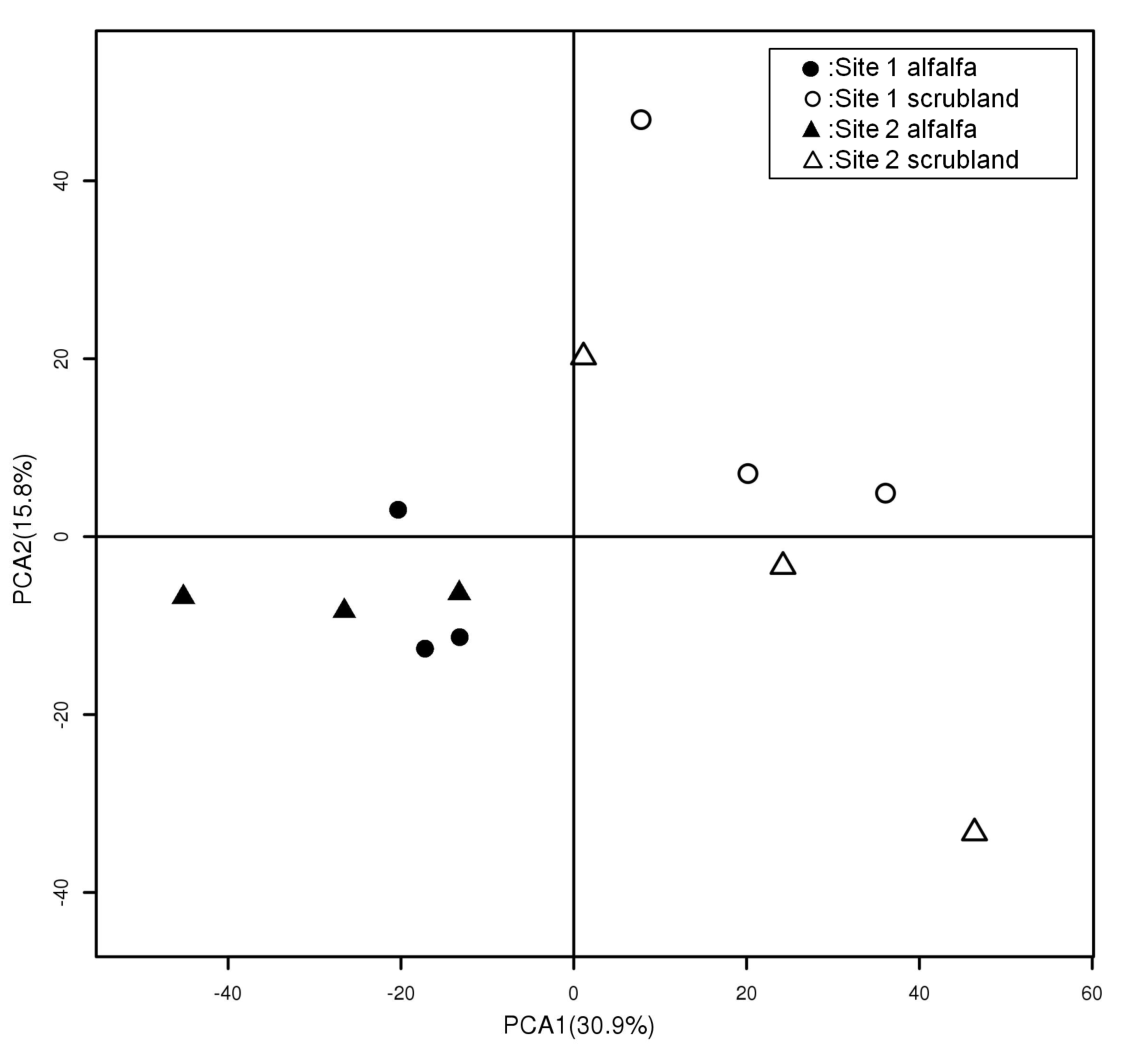

Supplement: Figure S5 — Principal component analysis of PhyloChip data for soils from different sites and land use. The first and second principal components explain 31% and 16% of total variance. (TIF) [file pone.0059497.s005.tif]

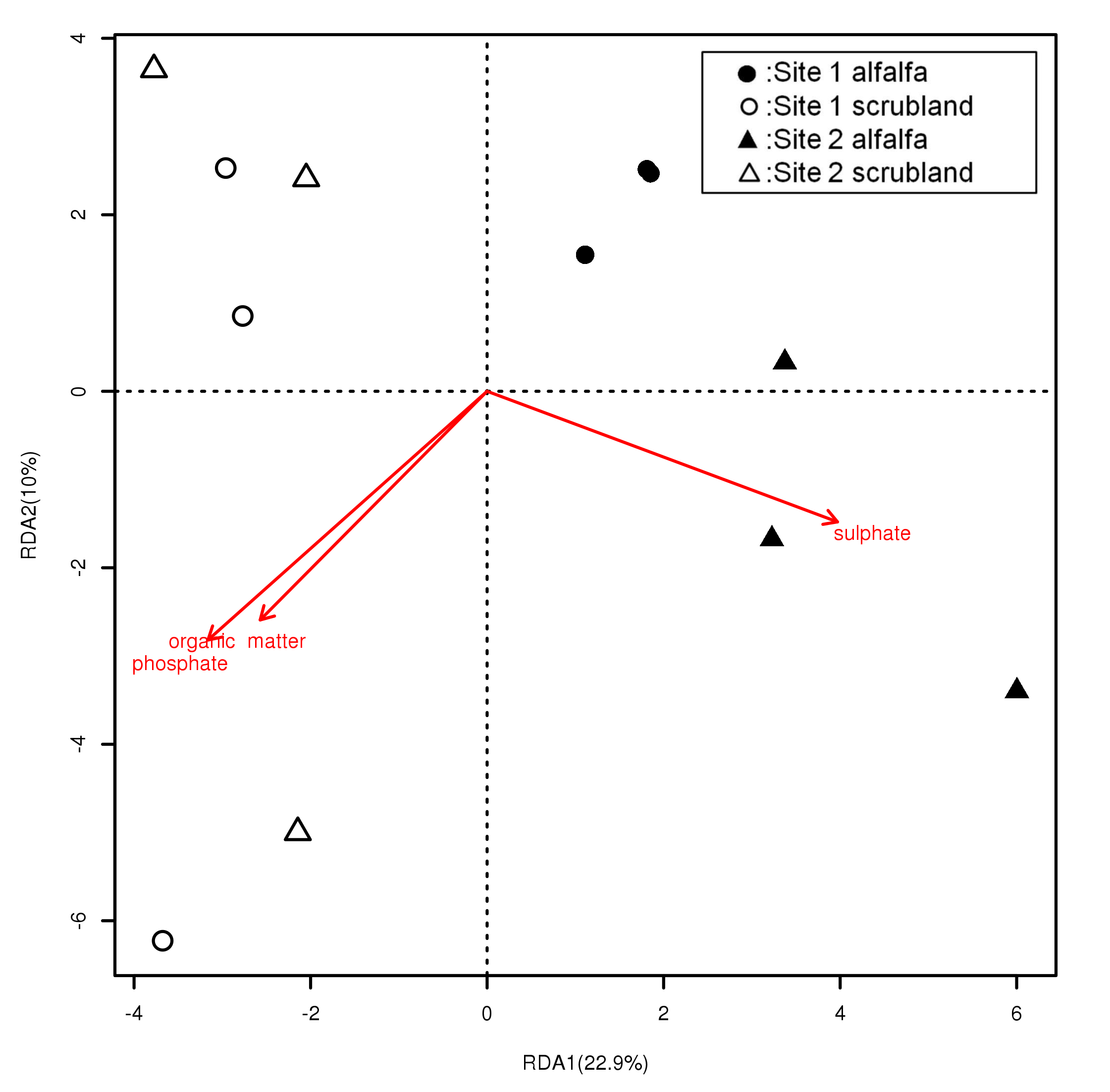

Supplement: Figure S6 — Redundancy analysis of the effect of discriminative soil parameters on the communities of Proteobacteria using the PhyloChip data. Numbers in brackets indicate the percent of the total variance explained by each axis. Only these soil parameters which significantly (p<0.05 by 1000 times permutation tests) explained the proteobacterial community variation are shown. (TIFF) [file pone.0059497.s006.tiff]

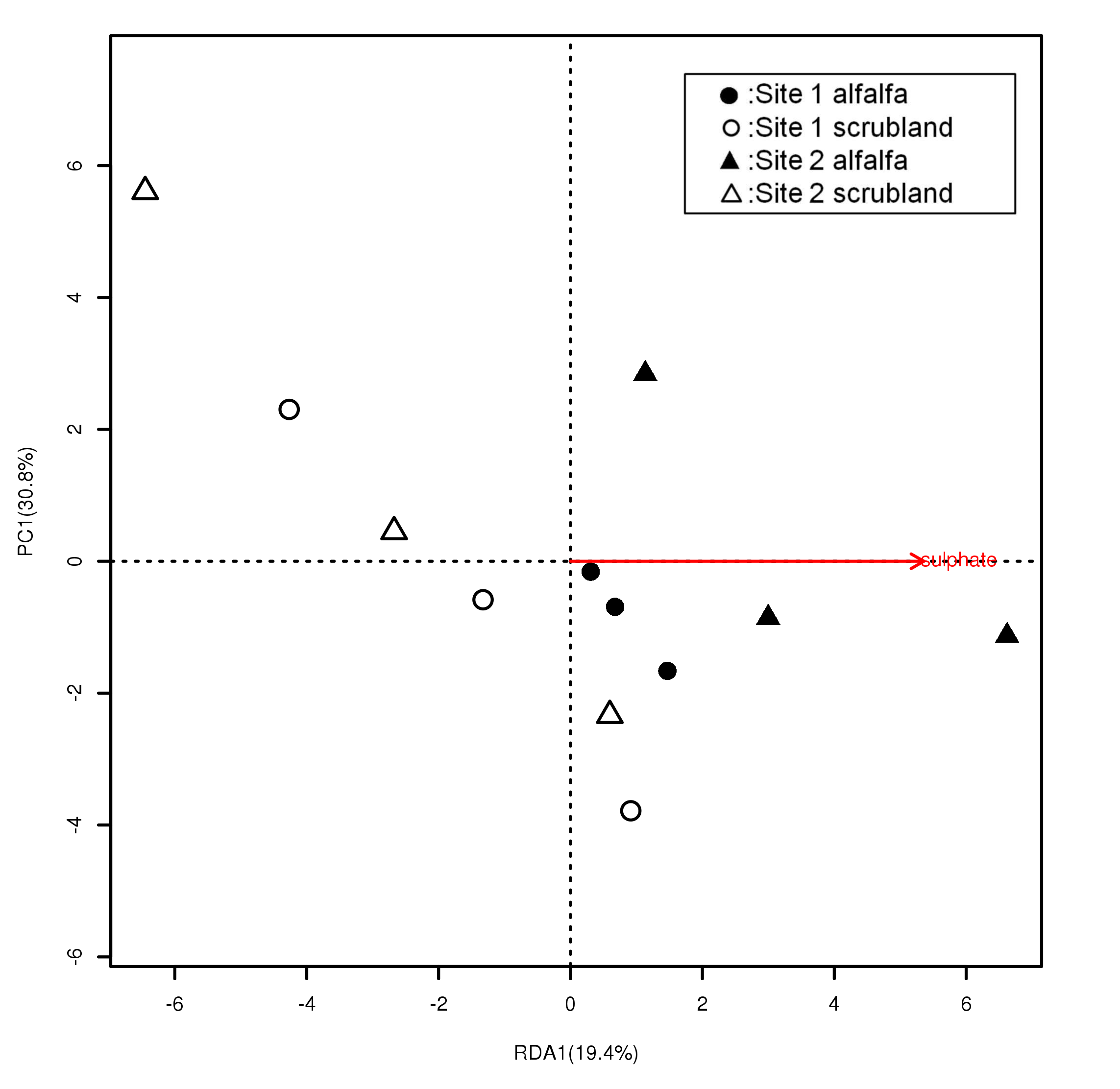

Supplement: Figure S7 — Redundancy analysis of the effect of discriminative soil parameters on the communities of Firmicutes using the PhyloChip data. Numbers in brackets indicate the percent of the total variance explained by each axis. Only the soil parameter which significantly (p<0.05 by 1000 times permutation tests) explained the variation of Firmicutes community is shown. (TIFF) [file pone.0059497.s007.tiff]

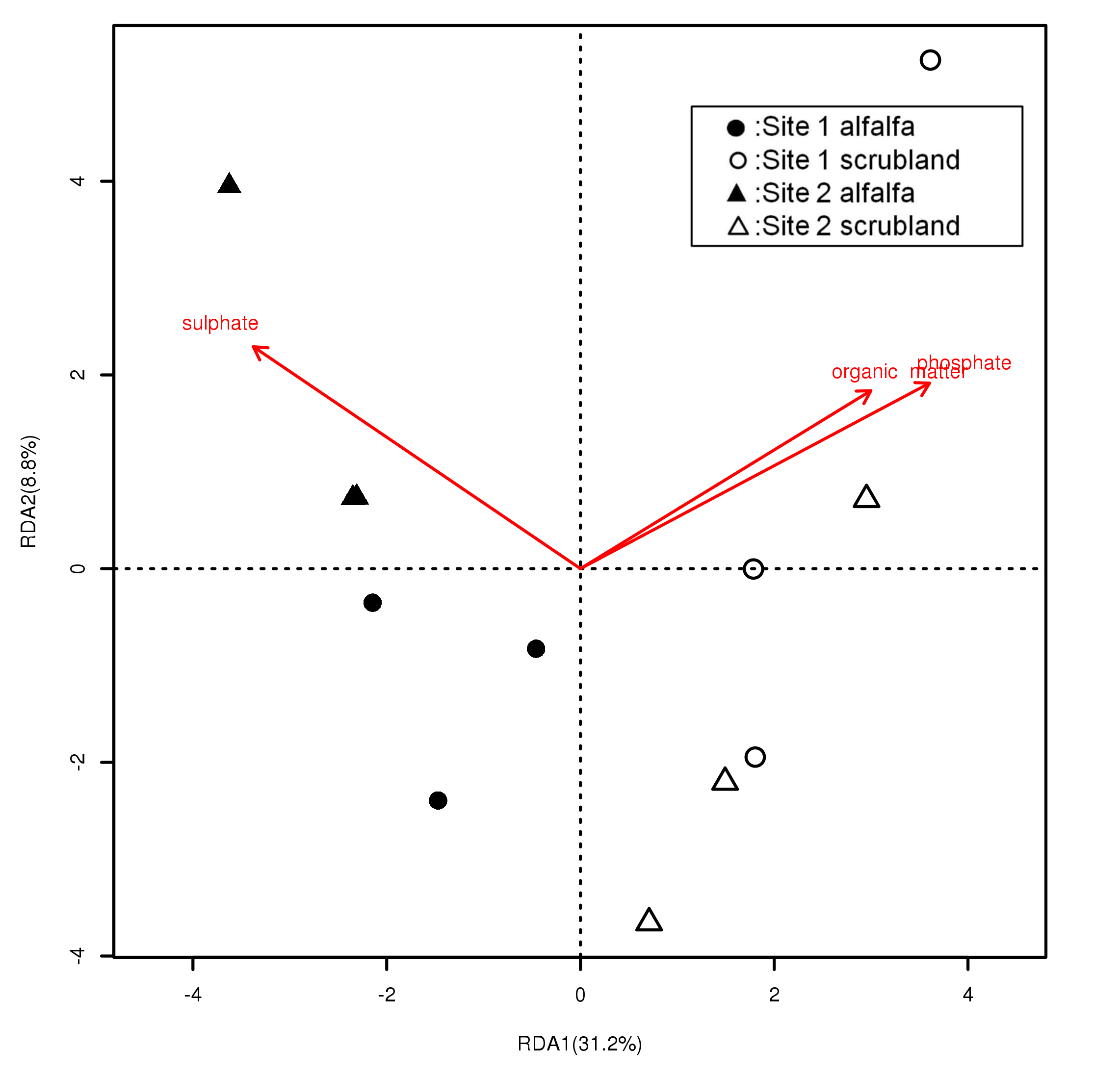

Supplement: Figure S8 — Redundancy analysis of the effect of discriminative soil parameters on the communities of Actinobacteria using the PhyloChip data. Numbers in brackets indicate the percent of the total variance explained by each axis. Only these soil parameters which could significantly (p<0.05 by 1000 times permutation tests) explained the actinobacterial community variationare shown. (TIFF) [file pone.0059497.s008.tiff]

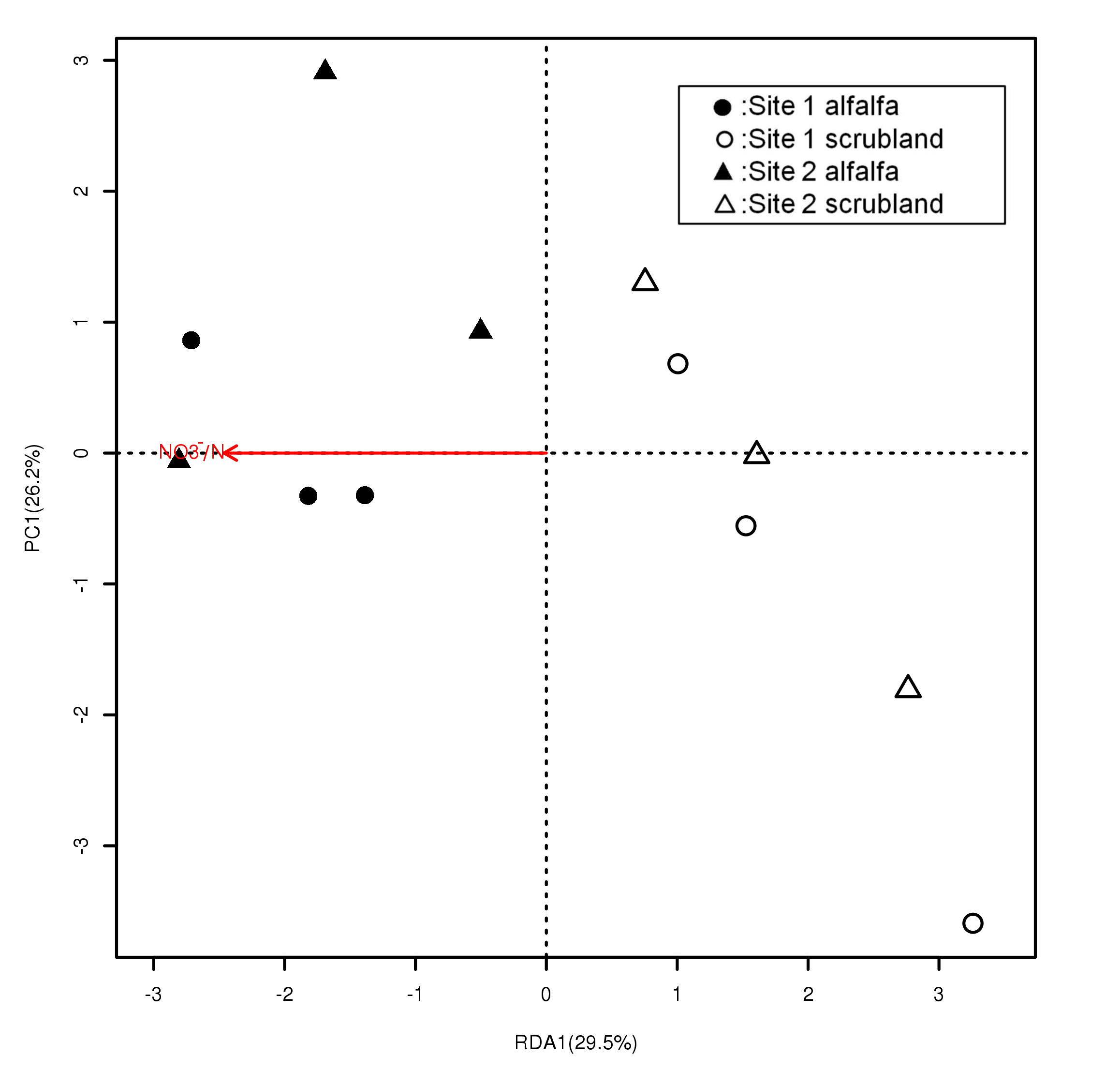

Supplement: Figure S9 — Redundancy analysis of the effect of discriminative soil parameters on the communities of Acidobacteria using the PhyloChip data. Numbers in brackets indicate the percent of the total variance explained by each axis. Only the soil parameters which significantly (p<0.05 by 1000 times permutation tests) explained the acidobacterial community variation is shown. (TIFF) [file pone.0059497.s009.tiff]

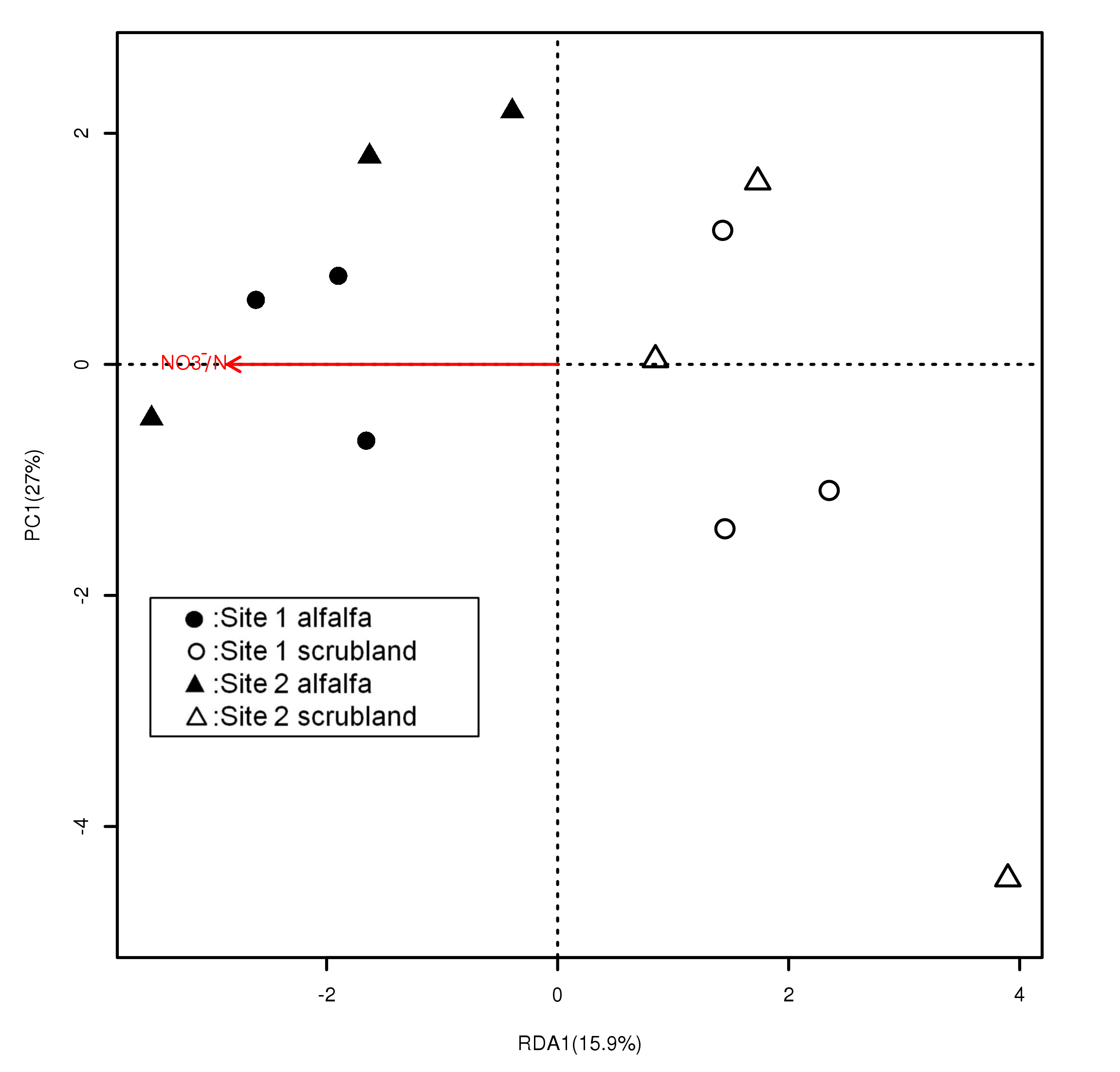

Supplement: Figure S10 — Redundancy analysis of the effect of discriminative soil parameters on the communities of Bacteroidetes using the PhyloChip data. Numbers in brackets indicate the percent of the total variance explained by each axis. Only the soil parameters which significantly (p<0.05 by 1000 times permutation tests) explained the community variation of Bacteroidetes is shown. (TIFF) [file pone.0059497.s010.tiff]

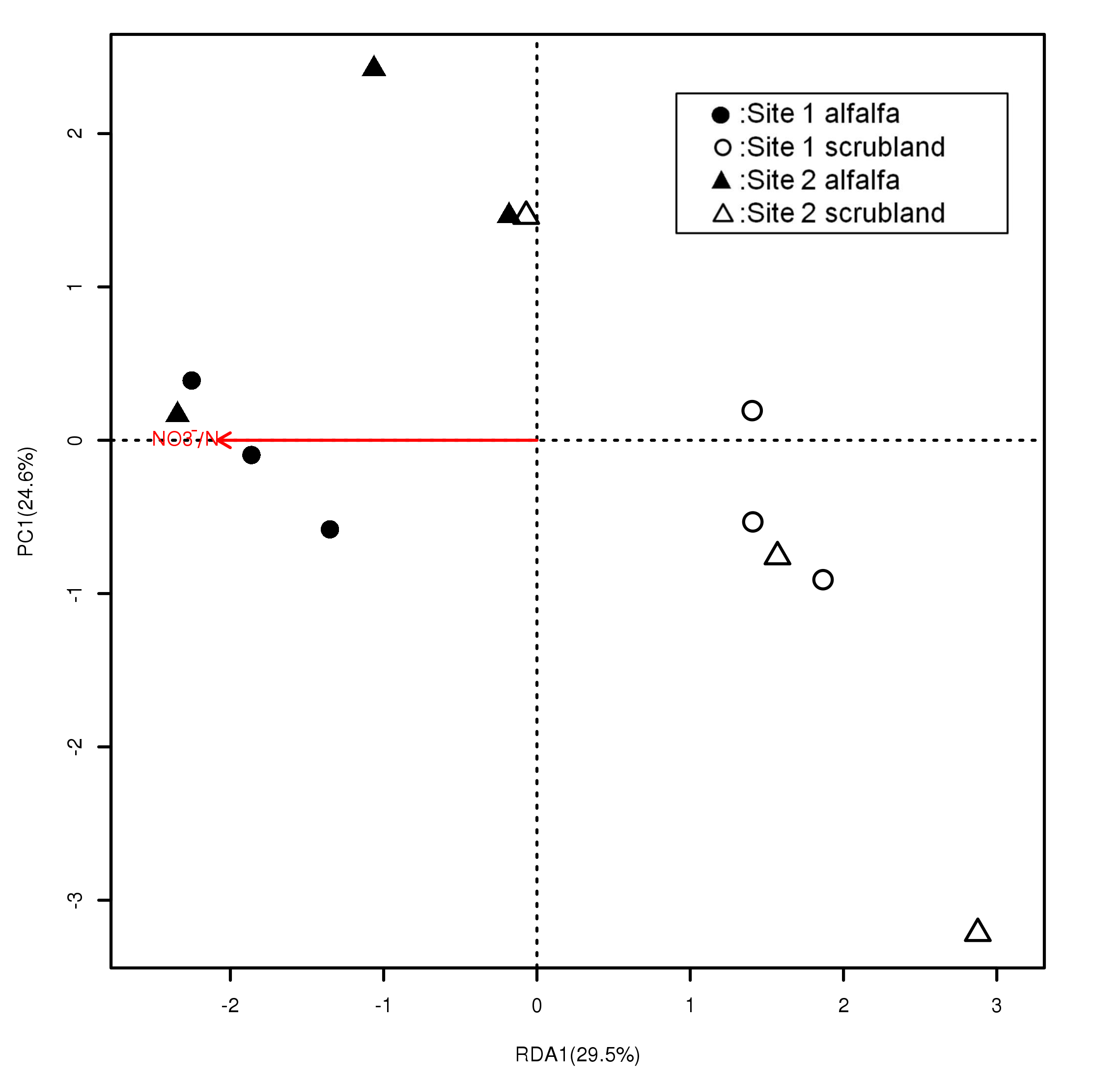

Supplement: Figure S11 — Redundancy analysis of the effect of discriminative soil parameters on the communities of Chloroflexi using the PhyloChip data. Numbers in brackets indicate the percent of the total variance explained by each axis. Only the soil parameter which significantly (p<0.05 by 1000 times permutation tests) explained the community variation of Chloroflexi is shown. (TIFF) [file pone.0059497.s011.tiff]

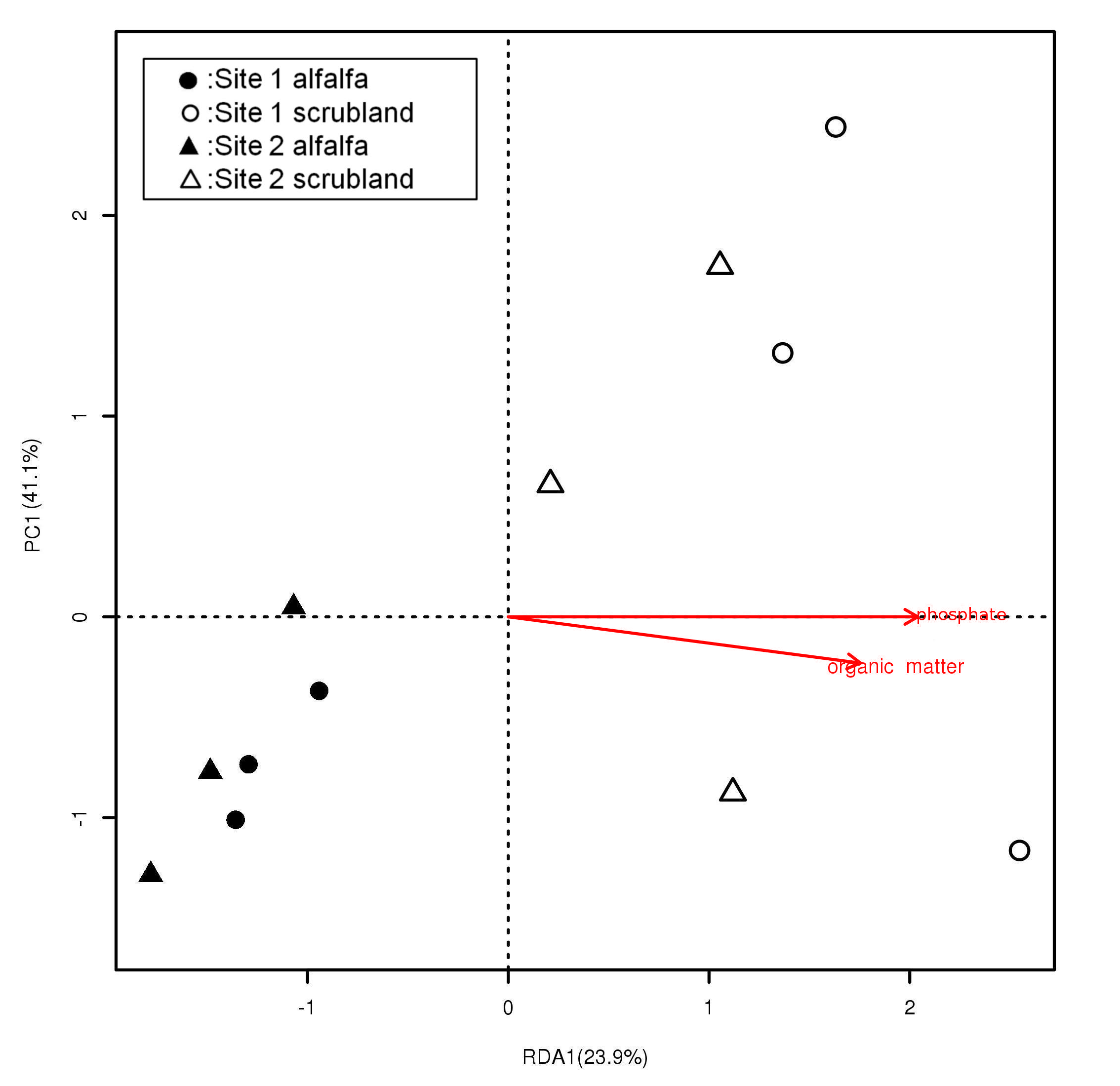

Supplement: Figure S12 — Redundancy analysis of the effect of discriminative soil parameters on the communities of Verrucomicrobia using the PhyloChip data. Numbers in brackets indicate the percent of the total variance explained by each axis. Only these soil parameters which significantly (p<0.05 by 1000 times permutation tests) explained the community variation of Verrucomicrobia are shown. (TIFF) [file pone.0059497.s012.tiff]

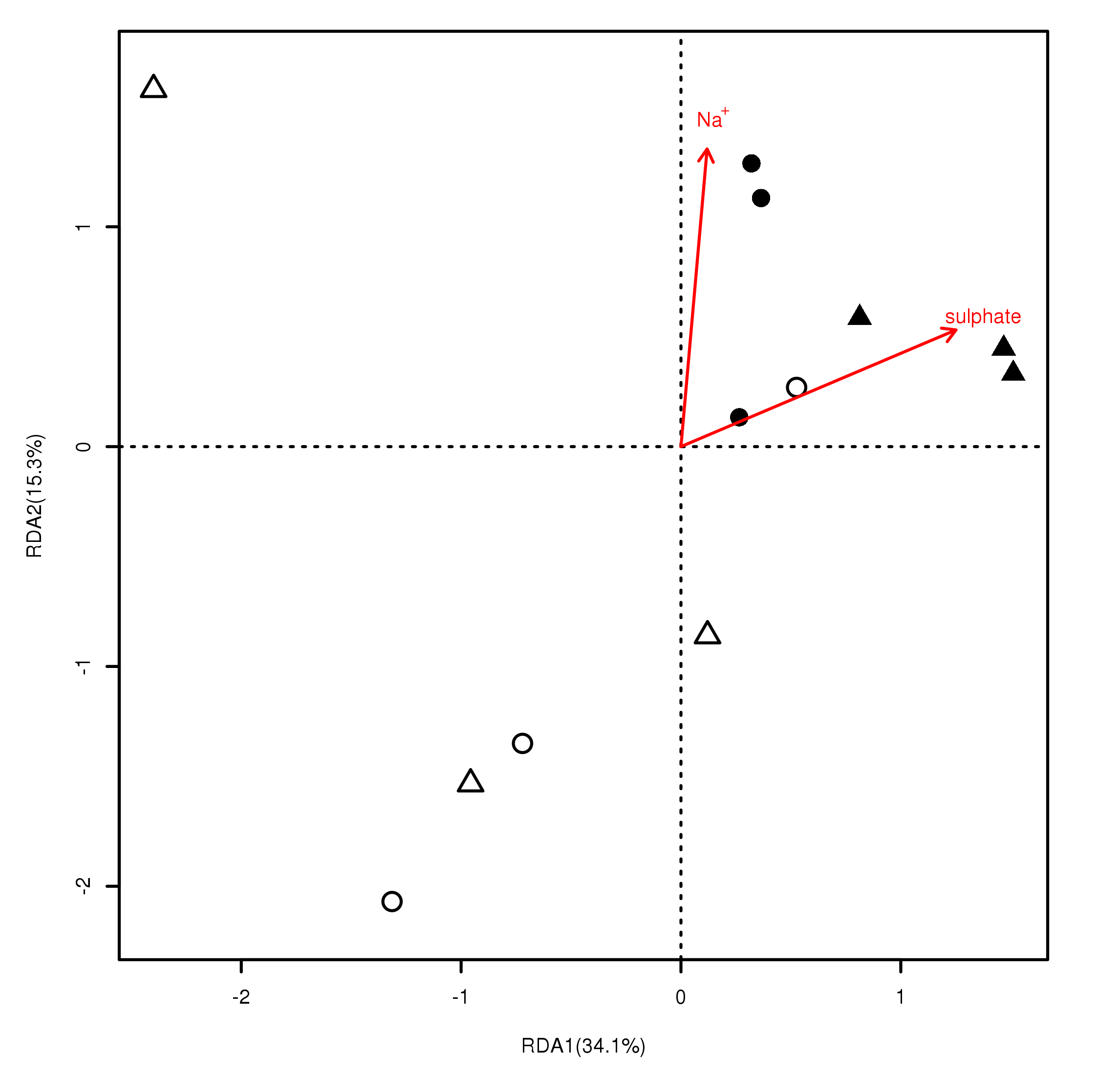

Supplement: Figure S13 — Redundancy analysis of the effect of discriminative soil parameters on the communities of Gemmatimonadetes using the PhyloChip data. Numbers in brackets indicate the percent of the total variance explained by each axis. Only these soil parameters which significantly (p<0.05 by 1000 times permutation tests) explained the community variation of Gemmatimonadetes are shown. (TIFF) [file pone.0059497.s013.tiff]

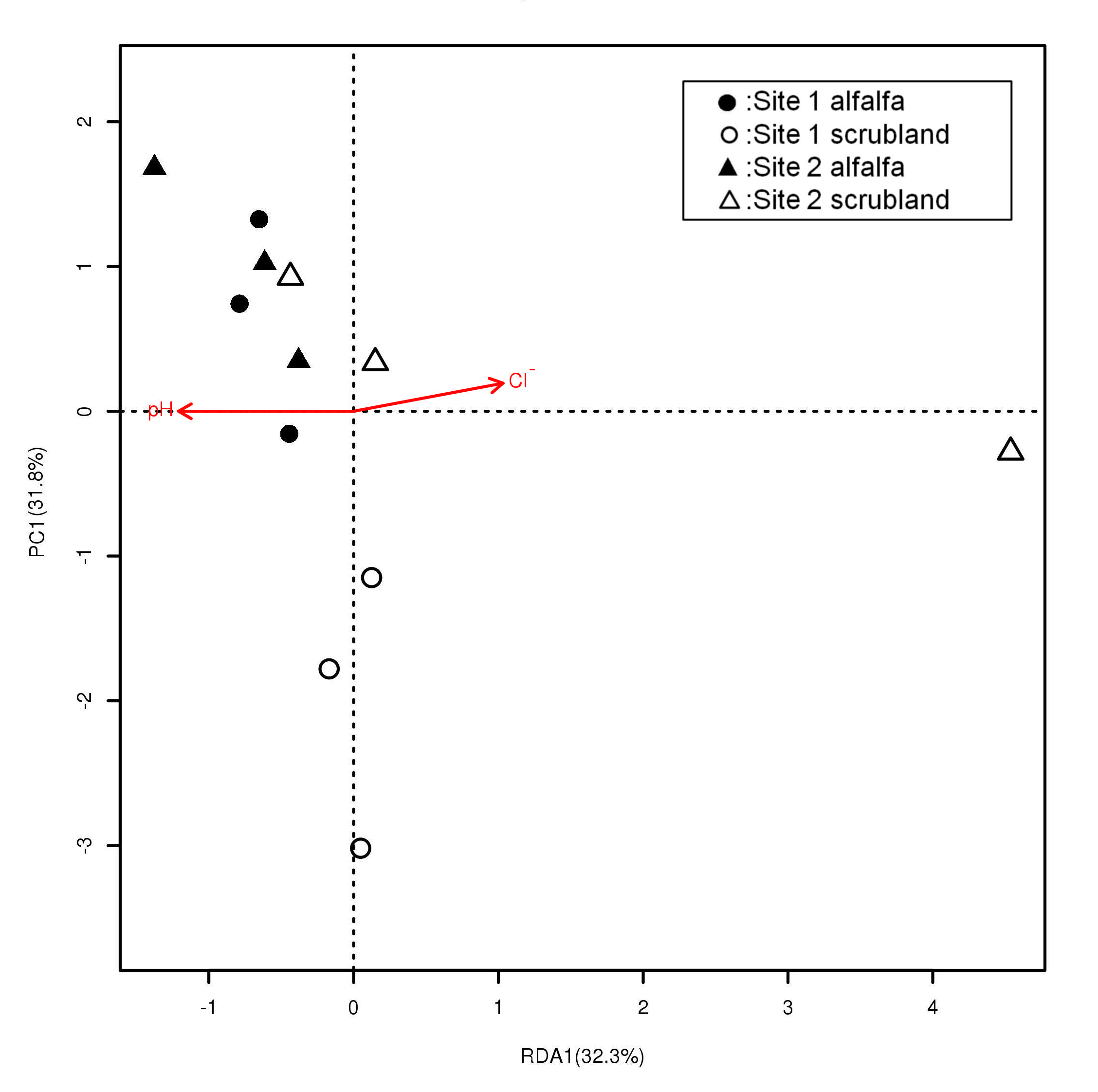

Supplement: Figure S14 — Redundancy analysis of the effect of soil parameters on the communities of Spirochaetes using the PhyloChip data. Numbers in brackets indicate the percent of the total variance explained by each axis. Only these soil parameters which significantly (p<0.05 by 1000 times permutation tests) explained the community variation of Spirochaetes are shown. (TIFF) [file pone.0059497.s014.tiff]
