# Supplementary material for: Changes of Soil Bacterial Diversity as a Consequence of Agricultural Land Use in a Semi-Arid Ecosystem
Source: PLoS One. 2013 Mar 20;8(3):e59497. doi: 10.1371/journal.pone.0059497 (PMC3603937; doi:10.1371/journal.pone.0059497)
Supplement: Table S1 — Primers used for DGGE analyses in the present study. (DOCX) [file pone.0059497.s015.docx]

Table S1. **Primers used for DGGE analyses in the present study.**

| Taxonic group | Primer pair | Primers | Sequences (5'–3') | Reference |
| --- | --- | --- | --- | --- |
| *Bacteria* | F984/R1378 | F984 | GCclamp-AACGCGAAGAACCTTAC | 28 |
|  |  | R1378 | CGGTGTGTACAAGGCCCGGGAACG |  |
| *Alphaproteobacteria* | F203/R1492 | F203 | CCGCATACGCCCTACGGGGGAAAGATTTAT | 29, 30 |
|  |  | R1492 | TACGGYTACCTTGTTACGACTT |  |
| *Betaproteobacteria* | F948/R1492 | F948 | CGCACAAGCGGTGGATGA | 29, 30 |
| *Actinobacteria* | F243/R1378 | F243 | GGATGAGCCCGCGGCCTA | 28 |

The GC clamp was *CGCCCGGGGCGCGCCCCGGGCGGGGCGGGGGCACGGGGGG.*
