# Supplementary material for: Changes of Soil Bacterial Diversity as a Consequence of Agricultural Land Use in a Semi-Arid Ecosystem
Source: PLoS One. 2013 Mar 20;8(3):e59497. doi: 10.1371/journal.pone.0059497 (PMC3603937; doi:10.1371/journal.pone.0059497)
Supplement: Table S2 — Numbers (average ± standard deviation)s of OTU detected for bulk soils from alfalfa field and scrubland at two sites. (DOCX) [file pone.0059497.s016.docx]

Table S2 Numbers (average ± standard deviation)s of OTU detected for bulk soils from alfalfa field and scrubland at two sites.

| **Phylum** | **Class** | **Order** | **Family** | Site 1 | |  | Site 2 | | Total ^a^ |
| --- | --- | --- | --- | --- | --- | --- | --- | --- | --- |
|  |  |  |  | Alfalfa | Scrubland |  | Alfalfa | Scrubland |  |
| *Proteobacteria* | *Alphaproteobacteria* | *Rhizobiales* | *Rhizobiaceae* | 15±0 | 14±1 |  | 16±2 | 13±1 | 18 |
|  |  |  | *Phyllobacteriaceae* | 10±1 | 11±2 |  | 11±2 | 10±2 | 15 |
|  |  | *Sphingomonadales* |  | 38±0 | 34±1 |  | 43±4 | 32±4 | 50 |
|  |  | *Rhodobacterales* |  | 22±10 | 20±4 |  | 42±3 | 17±3 | 50 |
|  | *Betaproteobacteria* | *Burkholderiales* | *Comamonadaceae* | 53±1 | 46±4 |  | 51±2 | 41±9 | 58 |
|  | *Gammaproteobacteria* | *Alteromonadales* |  | 41±4 | 25±6 |  | 45±4 | 29±19 | 57 |
|  |  | *Pseudomonadales* | *Pseudomonadaceae* | 34±3 | 12±6 |  | 27±12 | 11±8 | 39 |
|  |  | *Legionellales* |  | 9±0 | 8±0 |  | 10±1 | 8±1 | 11 |
|  | *Deltaproteobacteria* | *Desulfobacterales* |  | 26±1 | 28±1 |  | 27±1 | 26±3 | 30 |
|  |  | *Desulfovibrionales* |  | 13±0 | 14±2 |  | 13±1 | 12±1 | 16 |
|  |  | *Syntrophobacterales* |  | 12±0.6 | 12±1 |  | 12±1 | 10±1.5 | 14 |
| *Firmicutes* | *Clostridia* |  |  | 151±3 | 152±10 |  | 145±6 | 136±9 | 177 |
| *Actinobacteria* | *Actinobacteria* | Acidimicrobiales |  | 13±1 | 15±2 |  | 14±2 | 11±3 | 18 |
|  |  | *Actinomycetales* | *Microbacteriaceae* | 18±1 | 16±1 |  | 18±3 | 12±1 | 23 |
|  |  |  | *Micromonosporaceae* | 19±1 | 19±1 |  | 19±1 | 18±1 | 22 |
|  |  |  | *Mycobacteriaceae* | 19±0 | 21±0 |  | 19±0 | 19±1 | 21 |
|  |  |  | *Micrococcaceae* | 13±1 | 14±1 |  | 15±2 | 13±0 | 17 |
|  |  |  | *Nocardiaceae* | 15±1 | 15±1 |  | 15±1 | 14±1 | 16 |
|  |  |  | *Pseudonocardiaceae* | 5±1 | 8±3 |  | 6±2 | 5±1 | 12 |
|  |  |  | *Cellulomonadaceae* | 9±1 | 9±0 |  | 10±1 | 9±1 | 11 |
|  |  | *Rubrobacterales* |  | 13±2 | 17±2 |  | 13±2 | 13±2 | 20 |
| *Acidobacteria* | *Acidobacteria* | *Acidobacteriales* | *Acidobacteriaceae* | 32±0 | 32±2 |  | 31±1 | 32±2 | 37 |
| *Bacteroidetes* |  |  |  | 93±5 | 81±6 |  | 93±8 | 68±13 | 121 |
| *Chloroflexi* |  |  |  | 35±2 | 40±2 |  | 38±2 | 35±1 | 47 |
| *Spirochaetes* |  |  |  | 34±1 | 35±1 |  | 33±1 | 28±6 | 40 |
| *Verrucomicrobia* |  |  |  | 19±1 | 23±3 |  | 19±1 | 20±2 | 27 |
| *Gemmatimonadetes* |  |  |  | 7±1 | 9±1 |  | 9±1 | 8±2 | 9 |
| Bacteria (Total) ^b^ |  |  |  | 1709±21 | 1675±92 |  | 1743±44 | 1506±148 | 2243 |

Note: a: the total number of OTU detected belonging to each taxon; b the number of bacterial OTU detected for each treatment.
